# Author Correction: Defective internal allosteric network imparts dysfunctional ATP/substrate-binding cooperativity in oncogenic chimera of protein kinase A

**DOI:** 10.1038/s42003-021-02006-3

**Published:** 2021-04-06

**Authors:** Cristina Olivieri, Caitlin Walker, Adak Karamafrooz, Yingjie Wang, V. S. Manu, Fernando Porcelli, Donald K. Blumenthal, David D. Thomas, David A. Bernlohr, Sanford M. Simon, Susan S. Taylor, Gianluigi Veglia

**Affiliations:** 1 Department of Biochemistry, Molecular Biology, and Biophysics, University of Minnesota, Minneapolis, MN USA; 2 Chemistry, University of Minnesota, Minneapolis, MN USA; 3 DIBAF - University of Tuscia – Largo dell’ Università, Viterbo, Italy; 4 Department of Pharmacology and Toxicology, University of Utah, Salt Lake City, UT USA; 5 Laboratory of Cellular Biophysics, Rockefeller University, New York, NY USA; 6 Department of Chemistry and Biochemistry and Pharmacology, University of California at San Diego, La Jolla, CA USA; 7 Present Address: Shenzhen Bay Laboratory, Shenzhen, China

**Keywords:** Solution-state NMR, Solution-state NMR

Correction to: *Communications Biology* 10.1038/s42003-021-01819-6, published online 10 March 2021.

The original version of this Article contained an error in the spelling of the author Sanford M. Simon, which was incorrectly given as Simon M. Sandford.

This has now been corrected in both the PDF and HTML versions of the Article.

