# Supplementary material for: Defective internal allosteric network imparts dysfunctional ATP/substrate-binding cooperativity in oncogenic chimera of protein kinase A
Source: Commun Biol. 2021 Mar 10;4:321. doi: 10.1038/s42003-021-01819-6 (PMC7946884; doi:10.1038/s42003-021-01819-6)
Supplement: Supplementary file 2 — Supplementary Information [file 42003_2021_1819_MOESM2_ESM.pdf]

Supplementary Information

Defective Internal Allosteric Network Imparts Dysfunctional ATP/Substrate Binding Cooperativity in Oncogenic Chimera of Protein Kinase A

Cristina Olivieri[1†], Caitlin Walker[1†], Adak Karamafrooz[1†], Yingjie Wang[1,2,‡], Manu V.S.[1], Fernando Porcelli[3], Donald K. Blumenthal[4], David D. Thomas[1], David A. Bernlohr[1], Simon M. Sandford[5], Susan S. Taylor[6,7], and Gianluigi Veglia[1,2]*

[1]Department of Biochemistry, Molecular Biology, and Biophysics and [2]Chemistry, University of Minnesota, Minneapolis, MN 55455, USA

[3]DIBAF - University of Tuscia – Largo dell' Università, 01100 Viterbo, Italy

[4]Department of Pharmacology and Toxicology, University of Utah, Salt Lake City, UT 84112, USA

[5]Laboratory of Cellular Biophysics, Rockefeller University, New York, NY 10065, USA

[6]Department of Chemistry and Biochemistry and Pharmacology, University of California at San Diego, La Jolla, CA 92093, USA

[†]These authors contributed equally

‡ Present address: Shenzhen Bay Laboratory, Shenzhen 518055, China

*Corresponding Author:

Gianluigi Veglia
Department of Chemistry and Department of Biochemistry,
Molecular Biology, and Biophysics,
312 Church St. SE, Minneapolis, MN 55455
Telephone: (612) 625-0758
Fax: (612) 625-5780
E-mail: vegli001@umn.edu.

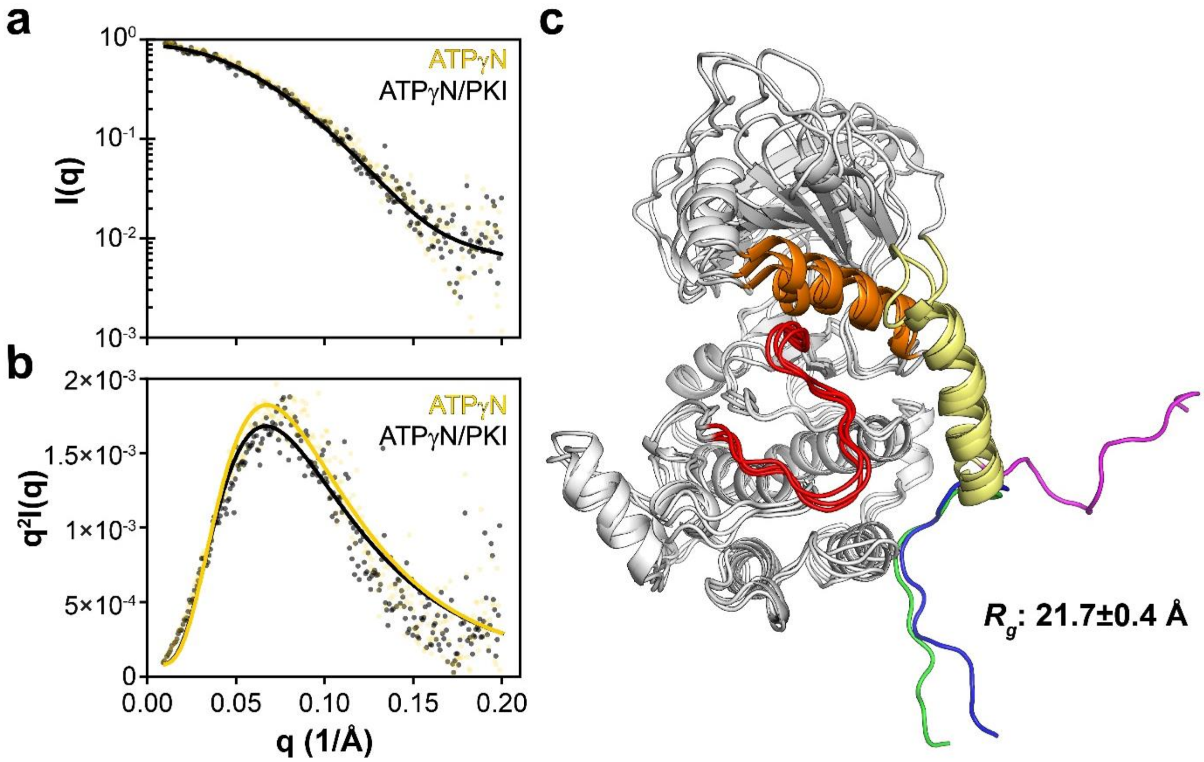

Supplementary Fig. 1: SAXS profiles for PKA-C^{WT}. **a** SAXS profiles of PKA-C^{WT} in the binary (ATPγN-bound, yellow) and ternary (ATPγN/PKI-bound, black) forms. Continuous lines show the fitting of the experimental SAXS data. **b** Corresponding Kratky plot of PKA-C^{WT} bound to ATPγN and ATPγN/PKI. **c** Overlay of selected snapshots of PKA-C^{WT}.

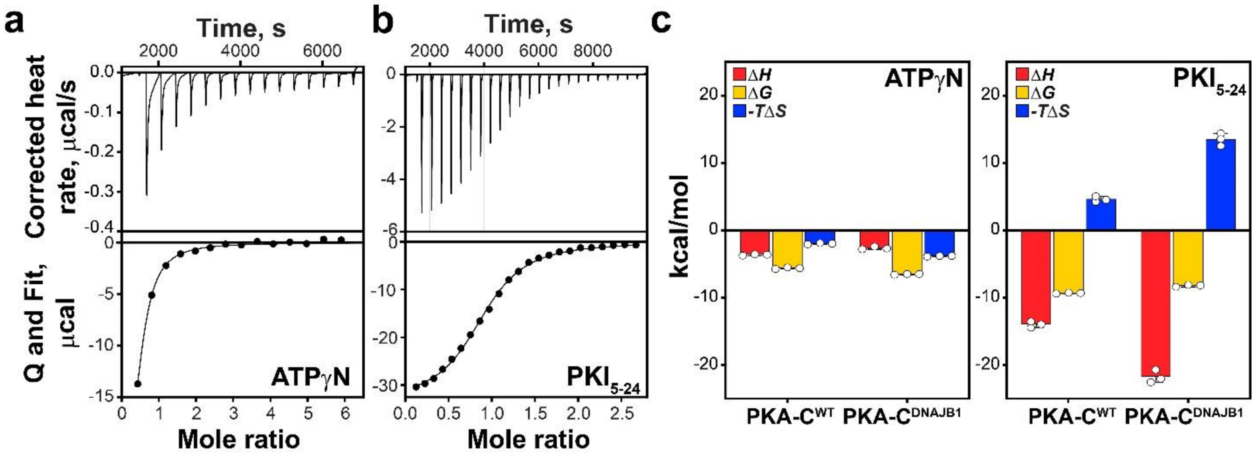

Supplementary Fig. 2: Thermodynamics of PKA-C^{DNAJB1} binding nucleotide and pseudo-substrate. Representative ITC thermographs of apo PKA-C^{DNAJB1} binding **a** ATPγN, and **b** PKI$_{5\text{-}24}$. Corresponding thermodynamic values are found in Supplementary Table 1, 2. **c** Graphical representation of the values of ΔH (red), ΔG (yellow), and -TΔS (blue). Errors are calculated as SD from triplicate measurements.

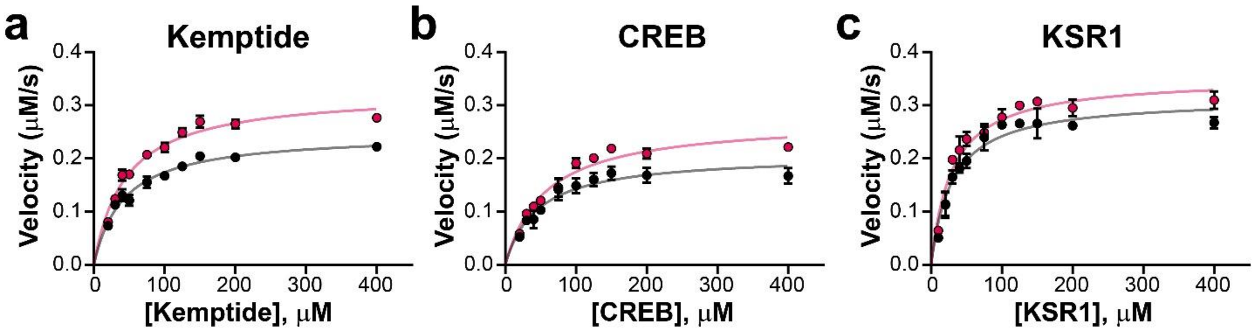

Supplementary Fig. 3: Steady state kinetics of phosphoryl transfer. Steady state phosphorylation kinetics of PKA-C^{WT} (black) and PKA-C^{DNAJB1} (pink) towards **a** Kemptide, **b** CREB, and **c** KSR1. See Supplementary Table 3 for corresponding kinetic parameters following fitting with the Michaelis-Menten equation.

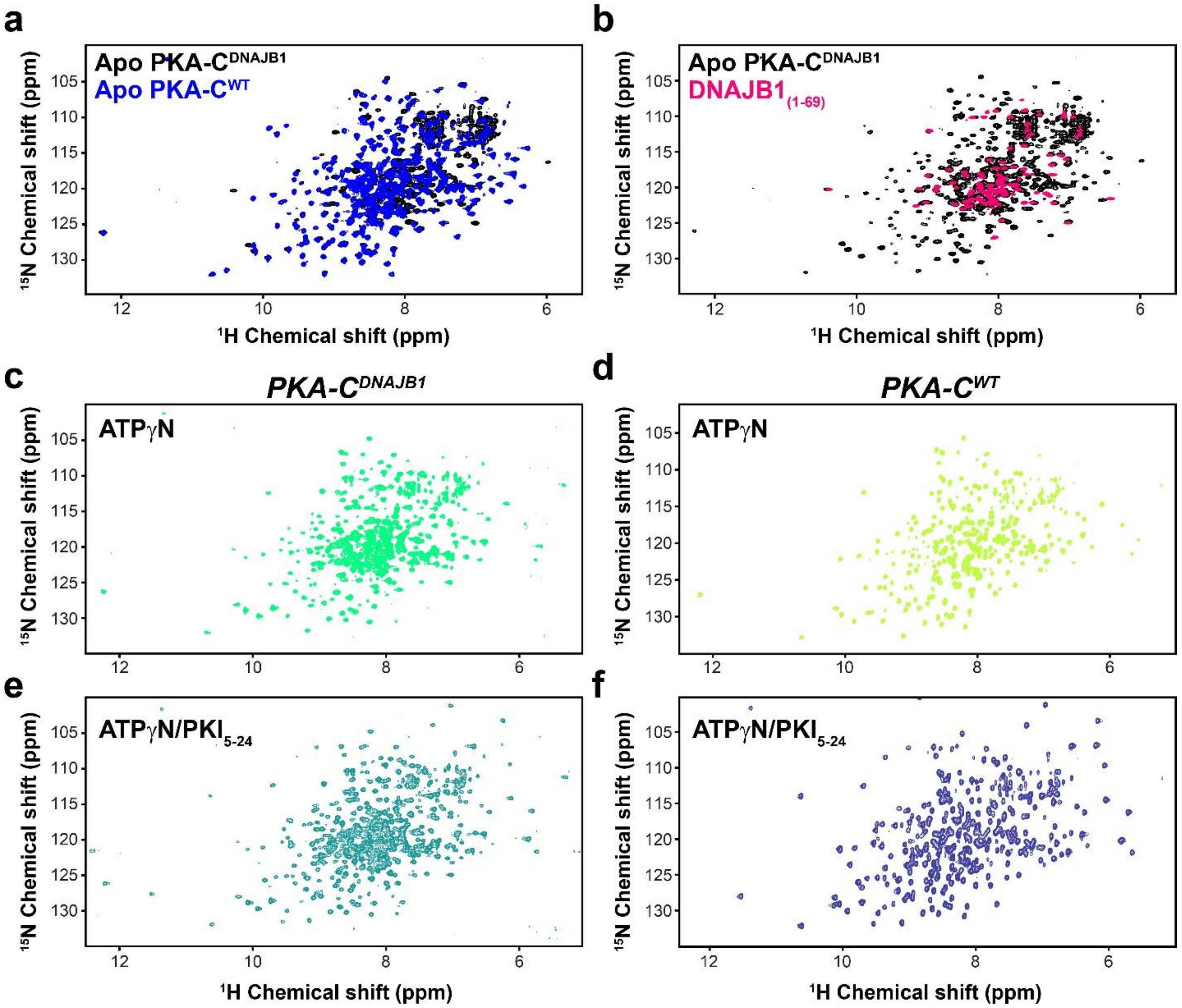

Supplementary Fig. 4: NMR amide fingerprints of PKA-C^{DNAJB1} and PKA-C^{WT}. [^{1}H,^{15}N]-TROSY-HSQC spectrum overlay of apo PKA-C^{DNAJB1} to **a** apo PKA-C^{WT} and **b** DNAJB1$_{(1-69)}$. [^{1}H,^{15}N]-TROSY-HSQC spectrum of PKA-C^{DNAJB1} bound to **c** ATPγN and **e** ATPγN/PKI$_{5-24}$, and of PKA-C^{WT} bound to **d** ATPγN and **f** ATPγN/PKI$_{5-24}$.

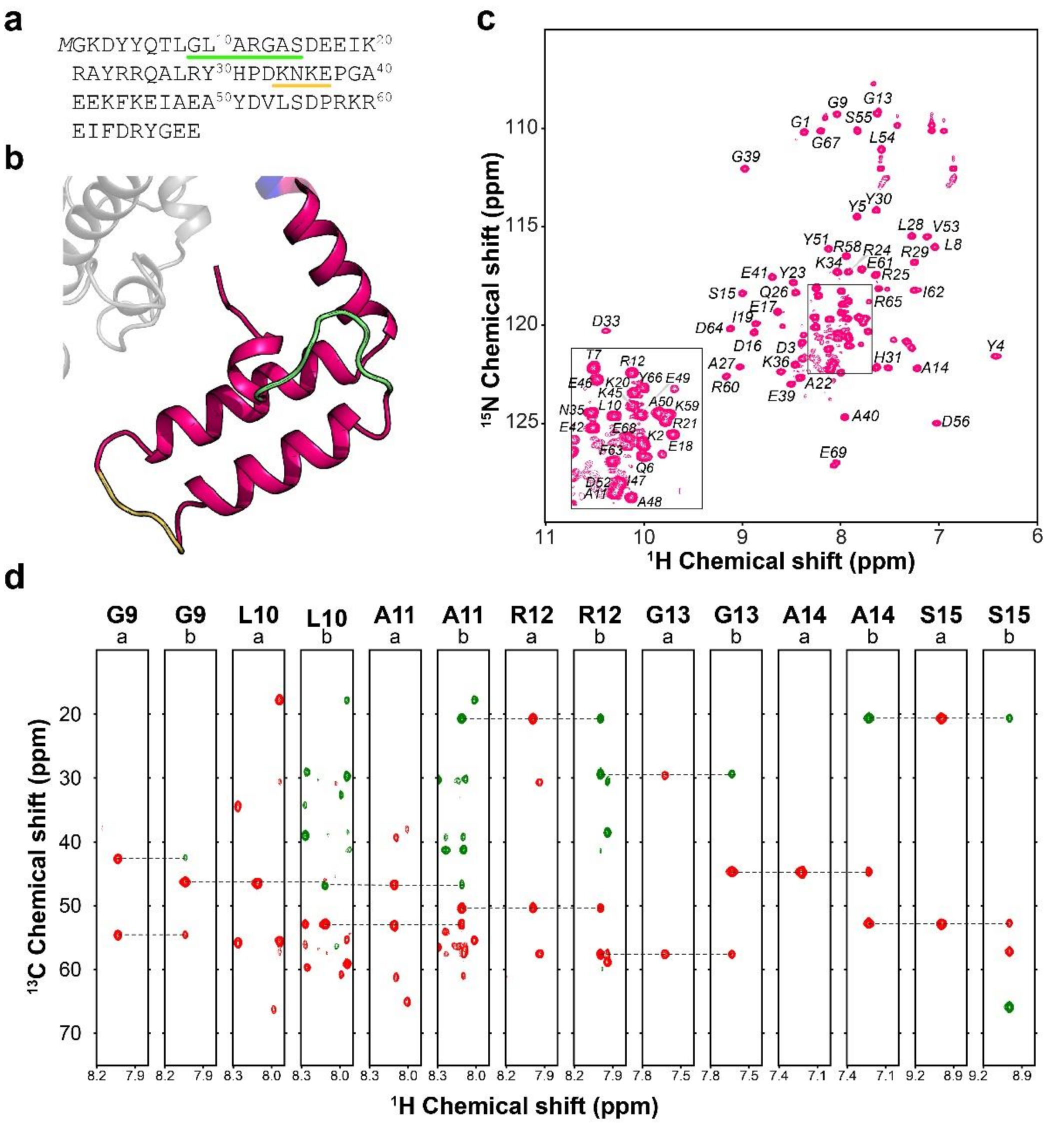

Supplementary Fig. 5: NMR backbone assignment of DNAJB1$_{1\text{-}69}$. **a** Primary sequence of DNAJB1 (Uniprot 25685). The sequence underlined in green are the residues which through-bound connectivity is reported in panel D. Residues that could not be assigned are underlined in yellow. **b** The three-dimensional structure of the J-domain (DNAJB1) of PKA-C^{DNAJB1} (PDB 4WB7). **c** [^{1}H,^{15}N]-Heteronuclear single quantum correlation (HSQC) spectrum of DNAJB1$_{1\text{-}69}$ with resonance assignment. **d** Series of strip plots from the CBCA(CO)HN (a) and HNCACB (b) experiments that illustrates the sequential connections between residue G9 and S15.

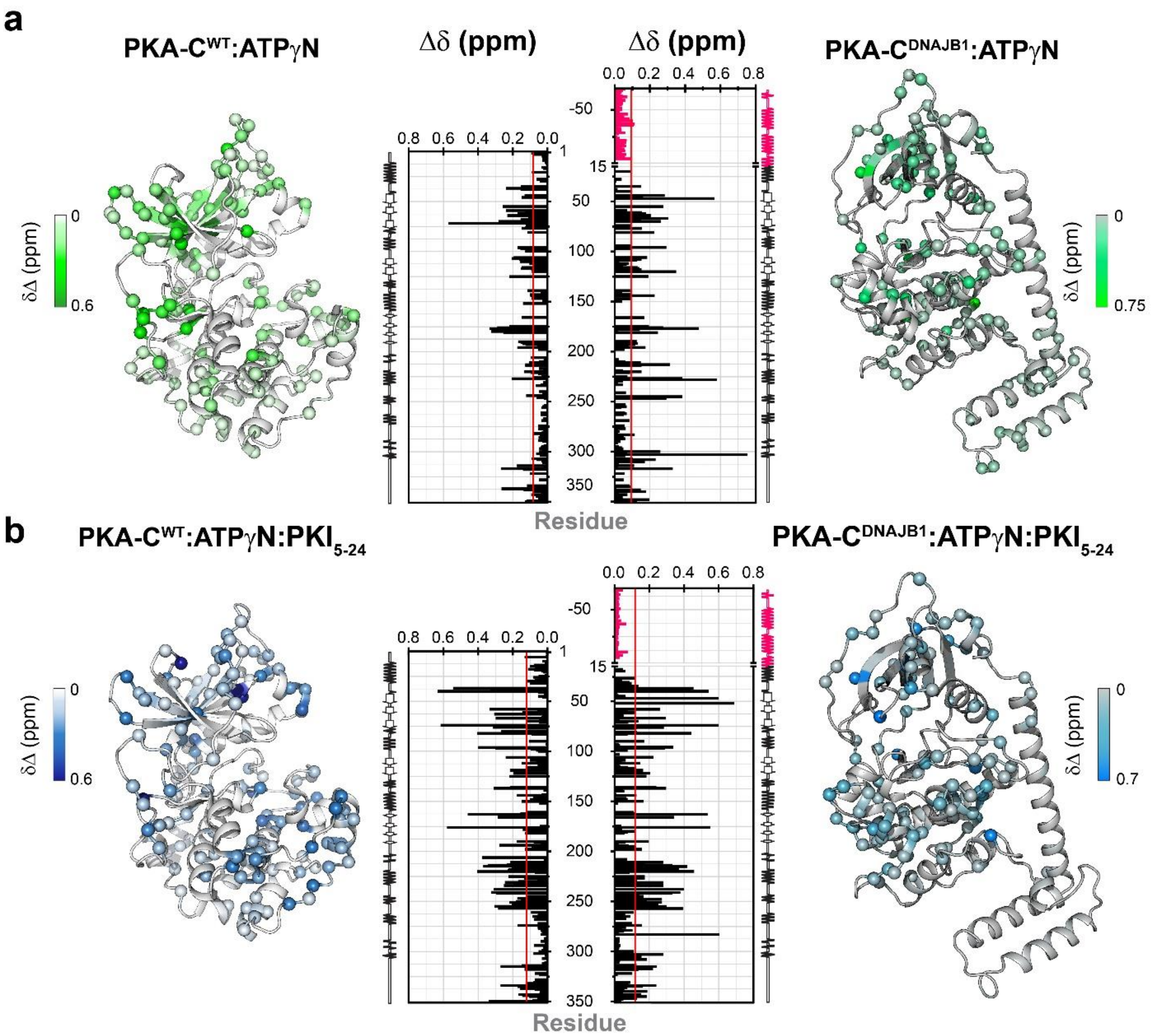

Supplementary Fig. 6: Chemical shift perturbations (CSP) observed upon ligand binding for PKA-C^{WT} and PKA-C^{DNAJB1}. Histograms show the combined ^{1}H-^{15}N chemical shift perturbations vs. residue for PKA-C^{WT} and PKA-C^{DNAJB1} in response to **a** ATPγN-binding and **b** ATPγN/$PKI_{5\text{-}24}$-binding. Each CSP is plotted on the structures of either PKA-C^{WT} (PDB: 4WB5) or PKA-C^{DNAJB1} (PDB: 4WB7). The red line on the histograms indicate one standard deviation from the average CSP. Note that the CSP values for PKA-C^{WT} are from [1].

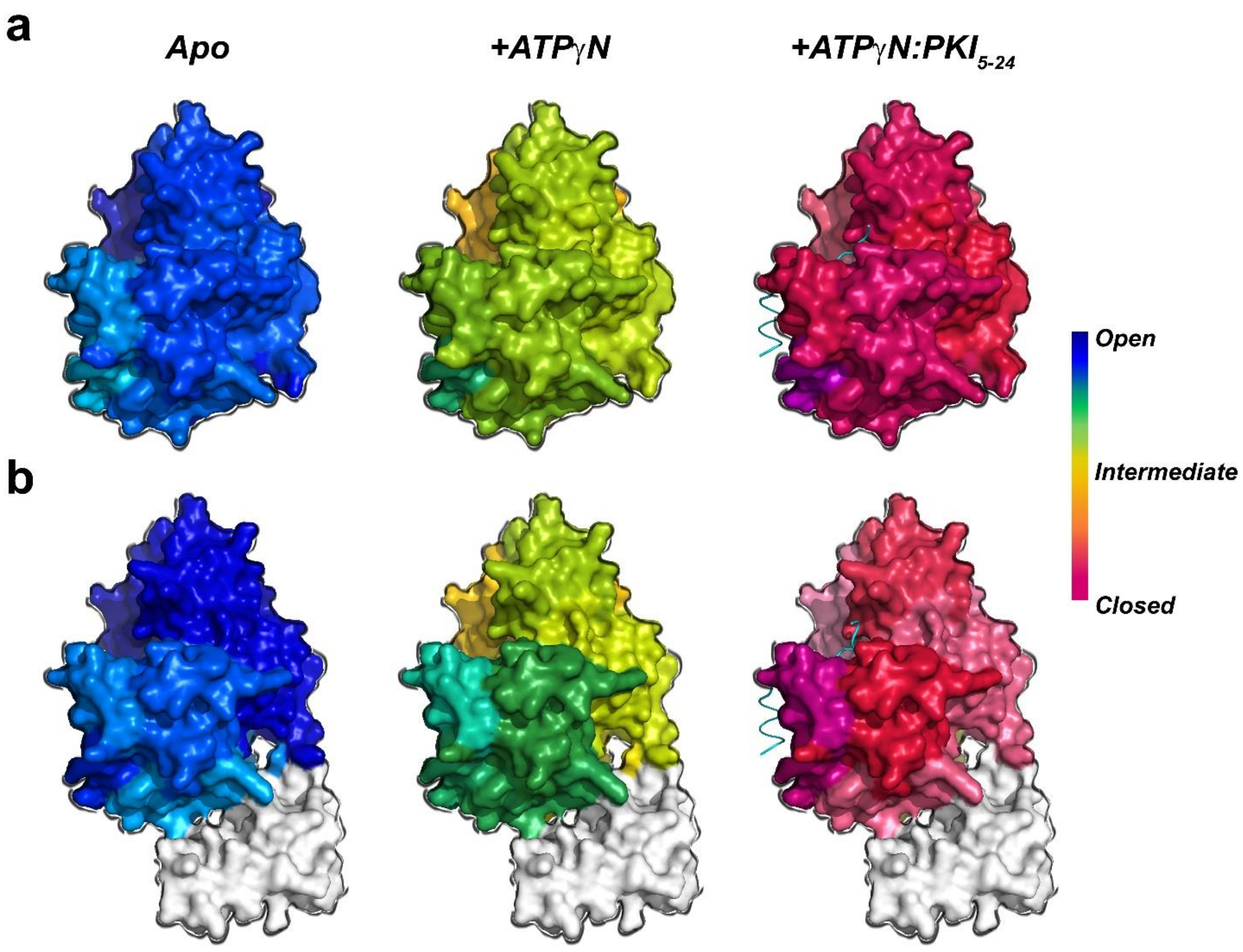

Supplementary Fig. 7: CONCISE scores of each individual community mapped onto the surface of **a** PKA-C^{WT} and **b** PKA-C^{DNAJB1}.

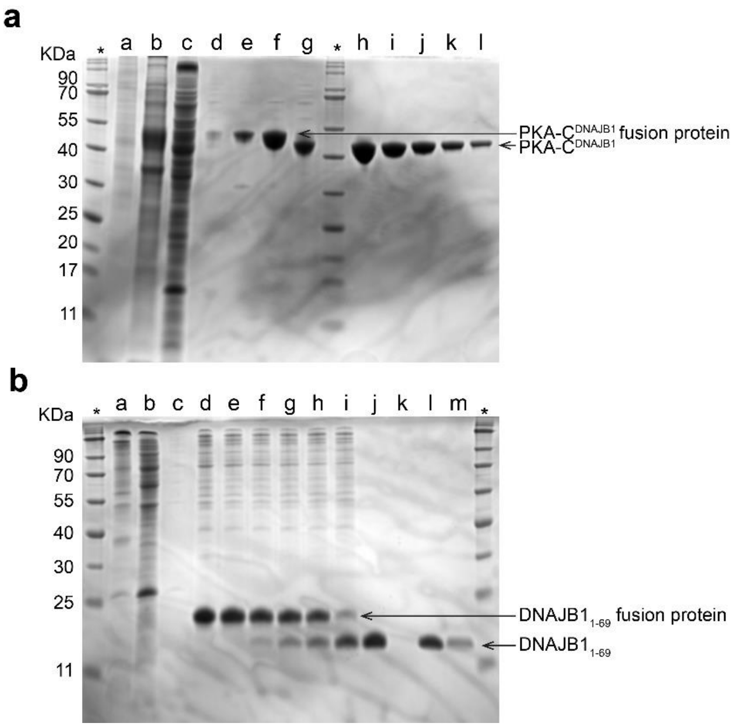

Supplementary Fig. 8: Isolation and purification of PKA-C^{DNAJB1} and DNAJB1$_{1-69}$. **a** Coomassie-stained 12% Acrylamide/bis-acrylamide SDS-PAGE of the expression and purification (a-g), and protein integrity test (i-k) of U-^{15}N PKA-C^{DNAJB1}. (*) BLUEstain™ Protein ladder (GoldBio), 11-245 kDa; (a) before induction of expression with 0.4 mM of IPTG; (b) after 12 hour expression; (c) Ni^{+}-NTA flow through; (d) wash 1; (e) wash 2; (f) elution; (g) after 18 hours of cleavage; (h) 15 μM of U-^{15}N *PKA-C^{DNAJB1}* used for the NMR titration; (i-l) serial dilutions of sample *h*. **b** Coomassie-stained 18% Acrylamide/bis-acrylamide SDS-PAGE of the purification of U-^{13}C/^{15}N DNAJB1$_{1-69}$. (*) BLUEstain™ Protein ladder (GoldBio), 11-245 kDa; (a) cell pellet; (b) Ni^{+}-NTA flow through; (c) wash; (d) elution; (e) before thrombin cleavage; (f) 1 hour into cleavage reaction; (g) 2 hours into cleavage reaction; (h) 3 hours into cleavage reaction; (i) 4 hours into cleavage reaction; (j) flow through from 10 kDa concentrator; (k) flow through from 3 kDa concentrator; (l) supernatant of 3 kDa concentrator; (m) NMR sample.

Supplementary Table 1: Changes in enthalpy, entropy, free energy, and dissociation constant of binding ATPγN for PKA-C^{WT} and PKA-C^{DNAJB1} derived from ITC experiments. All errors were calculated using triplicate measurements. Note that the values of K_d for PKA-C^{WT} are taken from Walker *et al.* [1].

	K_d (μM)	ΔG (kcal/mol)	ΔH (kcal/mol)	-TΔS (kcal/mol)
PKA-C^{WT}	83 ± 8	-5.61 ± 0.06	-3.6 ± 0.1	- 2.0 ± 0.1
PKA-C^{DNAJB1}	19 ± 4	-6.5 ± 0.1	-2.7 ± 0.2	-3.8 ± 0.1

Supplementary Table 2: Changes in enthalpy, entropy, free energy, and dissociation constant for the binding of $PKI_{5\text{-}24}$ to the apo and nucleotide-saturated forms of PKA-C^{WT} and PKA-C^{DNAJB1} derived from ITC experiments. All errors were calculated using triplicate measurements. Errors in σ were propagated from errors in K_d. N/A indicates the value is not applicable to the particular measurements. Note that the values of K_d for PKA-C^{WT} are taken from Walker *et al.* [1].

Binding of $PKI_{5\text{-}24}$ to apo forms of kinases					
	K_d (μM)	ΔG (kcal/mol)	ΔH (kcal/mol)	-TΔS (kcal/mol)	σ
PKA-C^{WT}	17 ± 2	-6.6 ± 0.1	-10.8 ± 0.5	4.2 ± 0.5	N/A
PKA-C^{DNAJB1}	9 ± 2	-6.9 ± 0.1	-20.1 ± 0.4	13.1 ± 0.4	N/A
Binding of $PKI_{5\text{-}24}$ to the ATPγN saturated forms of kinases					
	K_d (μM)	ΔG (kcal/mol)	ΔH (kcal/mol)	-TΔS (kcal/mol)	σ
PKA-C^{WT}	0.16 ± 0.02	-9.33 ± 0.07	-13.9 ± 0.5	4.6 ± 0.4	106 ± 18
PKA-C^{DNAJB1}	1.1 ± 0.2	-8.2 ± 0.1	-22 ± 1	14 ± 1	8 ± 2

Supplementary Table 3: Kinetic parameters of Kemptide, CREB, and KSR1 phosphorylation for PKA-C^{WT} and PKA-C^{DNAJB1}. Values for K_M and k_{cat} were obtained from a non-linear least-squares analysis of the concentration-dependent initial phosphorylation rates using a standard coupled enzyme activity assay (related to **Fig. 2b** and **Supplementary Fig. 3**). Error in k_{cat}/K_M was propagated from the error in K_M and k_{cat}.

Kemptide		
	PKA-C^{WT}	PKA-C^{DNAJB1}
V_{max} (μM/s)	0.25 ± 0.01	0.33 ± 0.01
K_M (μM)	42 ± 5	44 ± 6
k_{cat} (s^{-1})	11.4 ± 0.5	15 ± 0.5
k_{cat}/K_M	0.27 ± 0.03	0.34 ± 0.05

CREB		
	PKA-C^{WT}	PKA-C^{DNAJB1}
V_{max} (μM/s)	0.21 ± 0.01	0.27 ± 0.02
K_M (μM)	45 ± 8	56 ± 10
k_{cat} (s^{-1})	9.5 ± 0.5	12.3 ± 0.9
k_{cat}/K_M	0.21 ± 0.04	0.22 ± 0.04

KSR1		
	PKA-C^{WT}	PKA-C^{DNAJB1}
V_{max} (μM/s)	0.31 ± 0.01	0.35 ± 0.02
K_M (μM)	30 ± 5	29 ± 5
k_{cat} (s^{-1})	14.1 ± 0.5	15.9 ± 0.9
k_{cat}/K_M	0.47 ± 0.08	0.6 ± 0.1

Supplementary Table 4: CONCISE analysis of PKA-C^{WT} and PKA-C^{DNAJB1}. Value of % closed was calculated as a function of average PC scores. See the Methods section for calculation of $\Delta\Delta G$ based on CONCISE analysis.

	Average PC Score	% Closed	$\Delta\Delta G$ (kcal/mol)
PKA-C^{WT} Apo	-1.02	0%	0
PKA-C^{DNAJB1} Apo	-1.02	0%	0
PKA-C^{WT} ATPγN	-0.03	46%	-6.9
PKA-C^{DNAJB1} ATPγN	0.03	49%	-7.4
PKA-C^{WT} ADP	-0.06	45%	N/A
PKA-C^{DNAJB1} ADP	-0.02	47%	N/A
PKA-C^{WT} ATPγN/PKI$_{5-24}$	1.11	100%	-15.0
PKA-C^{DNAJB1} ATPγN/PKI$_{5-24}$	1.01	95%	-14.3

Supplementary References

1 Walker, C. *et al.* Cushing's syndrome driver mutation disrupts protein kinase A allosteric network, altering both regulation and substrate specificity. *Science Advances* **5**, eaaw9298, doi:10.1126/sciadv.aaw9298 (2019).
